# Primary Lymphoepithelioma-Like Carcinoma of the Prostate Gland: A Review of the Literature

**DOI:** 10.1155/2016/1876218

**Published:** 2016-01-11

**Authors:** Anthony Kodzo-Grey Venyo

**Affiliations:** North Manchester General Hospital, Department of Urology, Delaunay's Road, Crumpsall, Manchester, UK

## Abstract

*Background*. Primary lymphoepithelioma-like carcinoma of the prostate gland (PLELCP) is rare with hardly any information on its diagnostic features and biological behaviour.* Aim*. To review the literature.* Method*. Various Internet data bases were searched.* Literature Review*. PLELCP is extremely rare and there are hardly any pictures of the tumour involving the prostate; hence it would appear that clinicians would need to use their knowledge of the microscopic and immunohistochemical characteristics of the tumour in the nasopharynx and urinary bladder as diagnostic aid. PLELCP on microscopy mimics nasopharyngeal LELC. The LELC component of the tumour is characterized by indistinct cytoplasmic borders and a syncytial growth pattern. The stroma may be densely infiltrated by lymphoid cells admixed with some plasma cells and neutrophils and at times prominent infiltration of eosinophils. PLELCPs tend to have adenocarcinoma, either as the only pattern or with additional ductal components or adenosquamous carcinoma. PLELCPs stain positively with PSA, PSAP, AMACR/P504S, EMA, and cytokeratins AE1/AE3, 7, 8, and 20. There is no consensus on treatment of PLECP. The reported prognosis has been poor.* Conclusions*. PLELCPs should be entered into a multicenter trial to determine the biological behaviour and to find the best treatment option that would improve the prognosis.

## 1. Introduction

Globally primary lymphoepithelioma-like carcinoma of the prostate is extremely rare and the unaccustomed practitioner would perhaps have difficulty in its diagnosis in view of lack of literature on the disease. There is the likelihood that clinicians would be unfamiliar with the biological behaviour of the disease and its management. The ensuing review paper on primary lymphoepithelioma-like carcinoma of the prostate is divided into Section 4.1 which has summarized the disease and Section 4.2 which contains narrations and discussions from few cases of primary lymphoepithelioma-like carcinoma of prostate and some from cases of lymphoepithelioma-like carcinoma of the urinary bladder which may provide information on relevant lessons to be learnt on the management of primary lymphoepithelioma-like carcinoma of prostate.

## 2. Aim

The aim is to review the literature on primary lymphoepithelioma-like carcinoma of the prostate gland.

## 3. Method

Various Internet data bases were searched including PubMed; Google; Google Scholar; Educus; and Medline. The search words used were primary lymphoepithelioma-like carcinoma of prostate; lymphoepithelioma-like carcinoma of prostate; primary lymphoepithelioma-like cancer of prostate; lymphoepithelioma-like cancer of prostate. Surprisingly, even though there are a number of published data on lymphoepithelioma-like carcinoma elsewhere (in nonprostatic sites including the nasopharynx and urinary bladder) there was hardly any information on lymphoepithelioma-like carcinoma of the prostate. All information obtained from only 8 references were found suitable to use for the review paper.

## 4. Literature Review

### 4.1. Overview

#### 4.1.1. General Comment

Primary lymphoepithelioma-like carcinoma of the prostate gland is extremely rare [1].

#### 4.1.2. Ages

Lopez-Beltran et al. [2] reported LECCP in patients whose ages ranged from 69 years to 82 years.

#### 4.1.3. Presentation

- Lower urinary tract obstructive symptoms [2],
- raised serum levels of PSA [2],
- haematuria [2].

#### 4.1.4. Digital Rectal Examination Findings

There is no specific digital rectal examination finding of the prostate gland of patients who have lymphoepithelioma-like carcinoma that would differentiate them from those with other carcinomas of the prostate gland. Rectal examination in lymphoepithelioma-like carcinoma may reveal any of the following: a benign feeling prostate, a nodule in the prostate, an irregularity in the contour of the prostate, a mass in the prostate, or a hard feeling prostate gland.

#### 4.1.5. Diagnosis

Diagnosis of lymphoepithelioma-like carcinoma of the prostate can be made based upon the microscopic and immunohistochemical characteristics of the tumour specimens obtained at biopsy and resection of the prostate or of prostatectomy specimens. Nevertheless, primary lymphoepithelioma-like carcinoma of the prostate is so rare that there are hardly any figures or pictures that illustrate the microscopic and immunohistochemical characteristics of the tumour in the prostate. In view of this pathologists and clinicians would need to bear in mind the characteristic appearance of the tumour elsewhere (like in the nasopharynx or urinary bladder) to use as a guide otherwise perhaps the diagnosis might be missed as a result of unfamiliarity.

#### 4.1.6. Microscopic Features

See Figures 1, 2, 3, 4, 5, 6, 7, and 8 taken from [3] which shows microscopic and immunohistochemical staining characteristics of lymphoepithelioma-like carcinoma of urinary bladder which are usually similar to the findings in lymphoepithelioma-like carcinoma of prostate in the absence of images from the prostate.

Microscopic examination of prostate biopsy specimens in lymphoepithelioma-like cell carcinoma of prostate tends to show the following:

- Malignant epithelial components: these tend to be densely infiltrated by lymphoid cells [1].
- The tumours mimic nasopharyngeal lymphoepithelioma-like carcinoma [1].
- The tumours tend to have adenocarcinoma, either as the only pattern or with additional ductal components or adenosquamous carcinoma [2].
- The lymphoepithelioma-like carcinoma like component of the tumour tends to be characterized by indistinct cytoplasmic borders and a syncytial growth pattern. The stroma may be densely infiltrated by lymphoid cells admixed with some plasma cells and neutrophils and at times prominent infiltration of eosinophils [2].
- Gleason grading which is used for adenocarcinoma of prostate is not recommended for lymphoepithelioma-like cell carcinoma of prostate [1].

#### 4.1.7. Immunohistochemical Staining Characteristics

**Figure 4:**
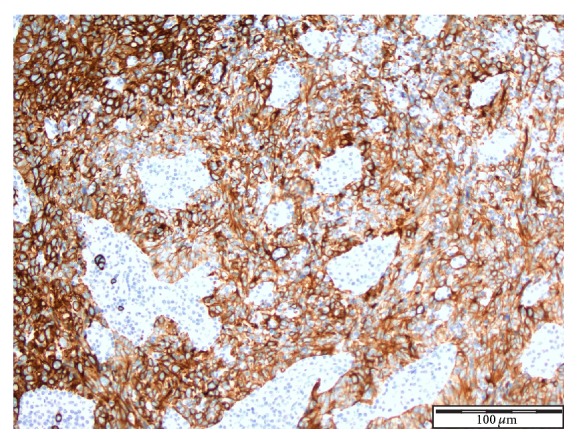
Lymphoepithelioma of urinary bladder-immunohistochemical staining for CK7. The neoplastic cells showing positive staining with CK7 in a background of inflammatory cells ×10 magnification reproduced from [3] (http://dx.doi.org/10.7707/hmj.v6i3.245) with permission from Hamdan Medical Journal which states that it is in the interest of both author and HMJ that any future uses of the article include its HMJ publication details to ensure that other people cite it correctly. Copy Right © 2013 The Authors: The Journal Compilation © 2013 Sheikh Hamdan Bin Rashid Al Maktoum Award for Medical Sciences Copy Right is still retained by the original source and future use and reproduction of the figures would require copy right permission from the original source via Hamdan Medical Journal [because of the scarcity of primary lymphoepithelioma of the prostate there is no figure on the disease available to the author; hence the use of the figure is related to the urinary bladder].

**Figure 5:**
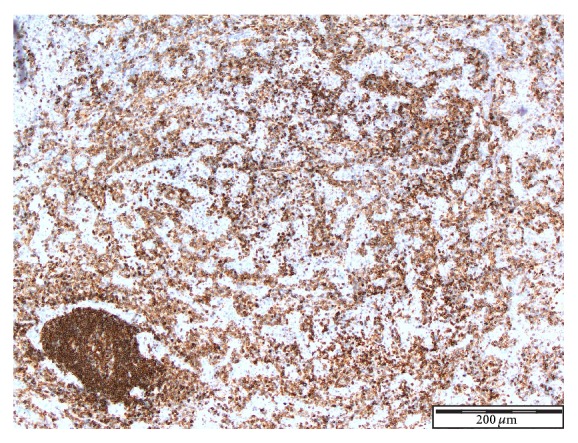
Immunohistochemical staining for LCA ×4 magnification. The background dense lymphoid infiltrate showing positive staining with LCA reproduced from [3] (http://dx.doi.org/10.7707/hmj.v6i3.245) with permission from Hamdan Medical Journal which states that it is in the interest of both author and HMJ that any future uses of the article include its HMJ publication details to ensure that other people cite it correctly. Copy Right © 2013 The Authors: The Journal Compilation © 2013 Sheikh Hamdan Bin Rashid Al Maktoum Award for Medical Sciences Copy Right is still retained by the original source and future use and reproduction of the figures would require copy right permission from the original source via Hamdan Medical Journal [because of the scarcity of primary lymphoepithelioma of the prostate there is no figure on the disease available to the author; hence the use of the figure is related to the urinary bladder].

**Figure 6:**
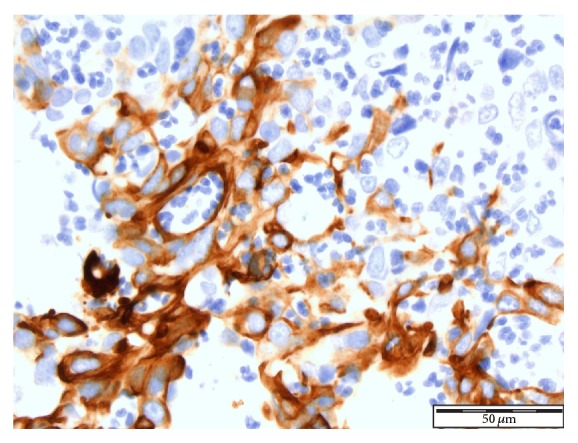
Immunohistochemical staining for CK20 ×40 magnification. Lymphoepithelioma-like carcinoma of the urinary bladder: the tumour cells show positive staining with CK20 reproduced from [3] (http://dx.doi.org/10.7707/hmj.v6i3.245) with permission from Hamdan Medical Journal which states that it is in the interest of both author and HMJ that any future uses of the article include its HMJ publication details to ensure that other people cite it correctly. Copy Right © 2013 The Authors: The Journal Compilation © 2013 Sheikh Hamdan Bin Rashid Al Maktoum Award for Medical Sciences Copy Right is still retained by the original source and future use and reproduction of the figures would require copy right permission from the original source via Hamdan Medical Journal [because of the scarcity of primary lymphoepithelioma of the prostate there is no figure on the disease available to the author; hence the use of the figure is related to the urinary bladder].

**Figure 7:**
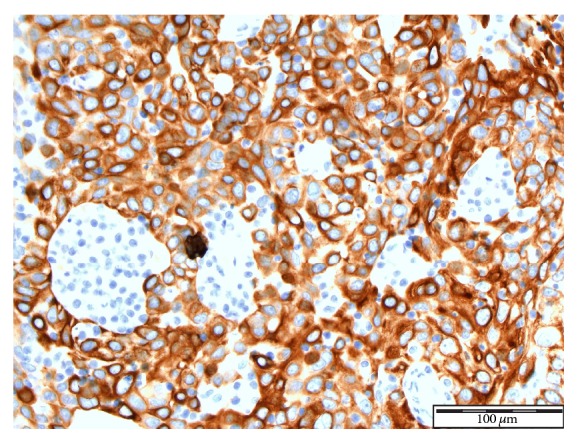
Immunohistochemical staining for 34bE12 (34*β*E12) ×20 magnification. Lymphoepithelioma-like carcinoma of urinary bladder: the neoplastic cells stain positive for 34bE12 (34*β*E12). Reproduced from [3] (http://dx.doi.org/10.7707/hmj.v6i3.245) with permission from Hamdan Medical Journal which states that it is in the interest of both author and HMJ that any future uses of the article include its HMJ publication details to ensure that other people cite it correctly. Copy Right © 2013 The Authors: The Journal Compilation © 2013 Sheikh Hamdan Bin Rashid Al Maktoum Award for Medical Sciences Copy Right is still retained by the original source and future use and reproduction of the figures would require copy right permission from the original source via Hamdan Medical Journal.

**Figure 8:**
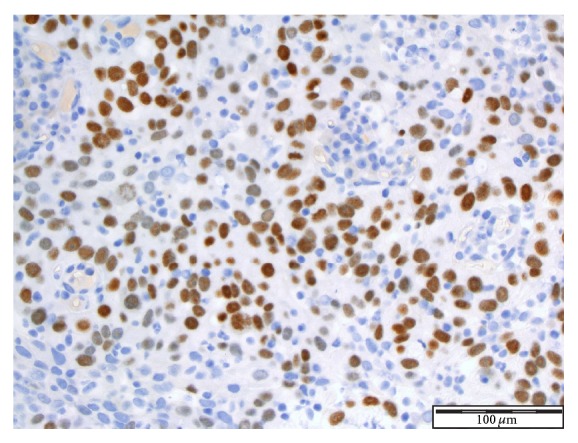
Immunohistochemical staining for p63 ×20 magnification. Lymphoepithelioma-like carcinoma of the urinary bladder: the tumour cells stain positive with p63 in a background of inflammatory cells reproduced from [3] (http://dx.doi.org/10.7707/hmj.v6i3.245) with permission from Hamdan Medical Journal which states that it is in the interest of both author and HMJ that any future uses of the article include its HMJ publication details to ensure that other people cite it correctly. Copy Right © 2013 The Authors: The Journal Compilation © 2013 Sheikh Hamdan Bin Rashid Al Maktoum Award for Medical Sciences Copy Right is still retained by the original source and future use and reproduction of the figures would require copy right permission from the original source via Hamdan Medical Journal [because of the scarcity of primary lymphoepithelioma of the prostate there is no figure on the disease available to the author; hence the use of the figure is related to the urinary bladder].

See Figures 4, 5, 6, 7, and 8 taken from [3] which shows microscopic and immunohistochemical staining characteristics of lymphoepithelioma-like carcinoma of urinary bladder which are usually similar to the findings in lymphoepithelioma-like carcinoma of prostate in the absence of images from the prostate.

*(1) Positive Stains*. Lymphoepithelioma-like cell carcinomas of the prostate gland tend to stain positively on immunohistochemistry with the following:

 PSA [1, 2],
 PSAP [1, 2],
 AMACR/P504S [1, 2],
 EMA [1, 2],
 cytokeratins AE1/AE3, 7, 8, and 20 [2].

The Ki-67 index of the lymphoepithelioma-like carcinoma ranged from 40% to 70% with a mean of 53% [2].

#### 4.1.8. DNA Ploidy

Lymphoepithelioma-like carcinomas of the prostate tend on DNA ploidy (flow cytometry study) to exhibit aneuploid peaks (aneuploidy) [2].

#### 4.1.9. Differential Diagnosis

Primary lymphoepithelioma-like carcinoma of the prostate gland should be differentiated from other malignancies involving the prostate including lymphoma and primary lymphoepithelioma-like variant of urinary bladder involving the prostate gland.

#### 4.1.10. Treatment

There is no consensus on the best treatment option for primary lymphoepithelioma-like carcinoma of the prostate in view of the limited number of cases that have been reported in the literature. Globally there is there is limited experience relating to the treatment of this disease. Nevertheless, treatment options include (a) radiotherapy (anecdotal reports would indicate that some lymphoepithelioma-like variants of urothelial carcinoma involving the urinary bladder respond to radiotherapy); (b) chemotherapy (anecdotal reports have indicated that lymphoepithelioma-like variants of urothelial carcinoma do respond to chemotherapy); (c) radical prostatectomy as performed for adenocarcinoma of prostate; (d) transurethral resection of prostate could be performed in cases of bladder outflow obstruction; (e) a combination of radiotherapy (external beam radiotherapy or brachytherapy) and systemic chemotherapy; (f) radical prostatectomy and radiotherapy; and (g) radical prostatectomy, which are all available treatment options which could be adopted. In view of the fact that there is no consensus opinion regarding treatment of the disease, perhaps a multicentre trial to determine the best option of treatment would be required. In view of the fact that conventional adenocarcinoma components may be present it may be that there may be a place for hormonal management to control the conventional adenocarcinoma elements of the tumour. If the tumour obstructs the ureter or ureters, there would be a need to insert a nephrostomy or a ureteric stent to preserve or improve impaired renal function. Other supportive management required for conventional adenocarcinoma of the prostate would be required in advanced cases. Kushida et al. [4] reported an 81-year-old man who underwent transurethral resection of bladder tumour and also received a total of 60 Gy of external beam radiotherapy for a T3N1M0 lymphoepithelioma-like variant of urothelial carcinoma who did not develop any recurrence at regular cystoscopies and on assessment of follow-up computed tomography scans but died of other causes 48 months later. Whether most patients with lymphoepithelioma-like carcinoma of prostate would respond to radiotherapy or not would hopefully be ascertained in the future. Williamson et al. [5] reported their study of 34 patients who had undergone treatment for lymphoepithelioma-like carcinoma of the urinary bladder and concluded that their findings supported the hypothesis that pure or predominant lymphoepithelioma-like carcinoma of the urinary bladder may be treated with transurethral resection and chemotherapy. Nevertheless, they recommended that a large-scale study with long-term follow-up would be required to better understand the biological behaviour of urinary bladder lymphoepithelioma-like carcinoma. Likewise, it would be argued that perhaps lymphoepithelioma-like cell carcinoma of prostate may respond to chemotherapy therefore patients with lymphoepithelioma-like carcinoma of the prostate should be treated with chemotherapy and they should also be entered into a large multicenter trial in order to confirm whether the patients would have a good long-term outcome or not.

#### 4.1.11. Outcome

Lymphoepithelioma-like carcinomas of the prostate gland tend to present at an advanced clinical stage and they tend to exhibit aggressive behaviour and tend to be associated with inferior outcome following treatment [2].

Whether or not radiotherapy or chemotherapy or chemoradiation would help to improve the prognosis would only be known as more cases are reported in the literature and as patients are entered into a global multicenter trial to determine the best treatment options.

### 4.2. Miscellaneous Narrations and Discussions from Some Reported Cases

Grignon [6] in 2004 stated that tumours that mimic lymphoepithelioma of the nasopharynx had been reported in numerous body sites including the prostate gland and these types of tumours are extremely rare and at the time of their publication only one case of lymphoepithelioma-like carcinoma, the first case of lymphoepithelioma-like carcinoma, had been reported and described by Botswick and Adlakha in 1994 [7]. They also stated that, in the case reported by Bostwick and Adlakha [7], the lymphoepithelioma-like carcinoma was admixed with a small acinar adenocarcinoma and that Amin et al. [8] had stated that the clinical significance of this morphology (morphology of lymphoepithelioma-like carcinoma) in the prostate was unknown; in view of this they would not recommend assignment of a Gleason grade.

Lopez-Beltran et al. [2] in 2009 summarized the clinicopathological characteristics of 5 cases of lymphoepithelioma-like carcinoma of the prostate which they stated is a rare variant of prostate cancer. They stated that the tumour is characterized by presence of malignant epithelial component densely infiltrated by lymphoid cells. All of the 5 patients had obstructive voiding symptoms and raised serum levels of prostate-specific antigen; one of the patients additionally had haematuria. They had a mean age of 76 years and their ages ranged from 69 years to 82 years. The diagnosis of lymphoepithelioma-like carcinoma of the prostate gland was made firstly by means of pathological examination of prostate specimens obtained following trans-urethral resection of prostate in 3 cases and radical prostatectomy in 2 other patients. With regard to one of the cases, the diagnosis of lymphoepithelioma-like carcinoma admixed with conventional acinar adenocarcinoma unexpectedly made at the time of transurethral resection for what had been adjudged to be benign prostatic hyperplasia. With regard to staging of the tumours, three of the patients had clinical stage T3 tumours, one of the patients had stage T4 tumour, and one patient had stage T1b tumour. Microscopic examination had revealed that all of the tumours contained lymphoepithelioma-like carcinoma, which constituted 10% to 90% of the entire tumour. All of the cases had adenocarcinoma, either as the only pattern in 5 cases or with an additional ductal component in 3 cases. Furthermore one of the cases also had in addition adenosquamous carcinoma. Immunohistological staining of the specimens showed that the lymphoepithelioma-like carcinoma was positive for prostate-specific antigen, prostate-specific acid phosphatase, alpha-methylacyl coenzyme A racemase, and epithelial membrane antigen; several cytokeratins (AE1/AE3, 7, 8, and 20 [rare cells]) were also immunoreactive (positively stained). The mean Ki-67 labelling index ranged from 40% to 70% with a mean of 53%. The p53 expression was low (10% to 20%). Lopez-Beltran et al. [2] also reported that the lymphoid component of the tumour mainly consisted of T cells with a minor subset of B cells, which were admixed with some dendritic cells and histiocytes as illustrated by S100 and CD68 immunoreactivity. Additionally, latent membrane protein I immunostaining and in situ hybridization for Epstein-Barr virus exhibited negative staining in all of the 5 lymphoepithelioma-like carcinoma cases. On flow cytometry, the DNA ploidy of the lymphoepithelioma-like carcinoma tumours exhibited DNA histograms with aneuploidy peaks (they exhibited aneuploidy). Lopez-Beltran et al. [2] also observed that the DNA ploidy of the concurrent adenocarcinoma exhibited DNA aneuploid peaks (aneuploidy) except in one case which exhibited diploid peak (diploidy). With regard to outcome, four patients died of their disease from 8 months to 26 months, but one patient was lost to follow-up whose outcome was not known. Lopez-Beltran et al. [2] made the ensuing summarizing conclusions: lymphoepithelioma-like carcinoma of the prostate does arise in aggressive carcinomas of the prostate at an advanced clinical stage. Morphological recognition and distinction from other lesions of the prostate and tumours with prominent lymphoid stroma is vital for its clinical management.

Williamson et al. [5] examined the clinicopathological characteristics of lymphoepithelioma-like variant of urothelial carcinoma that had involved the urinary tract of 34 patients. The features of lymphoepithelioma-like carcinoma they examined included light microscopy; immunohistochemistry for cytokeratin 7 (CK7), CK20, 34*β*E12, p53, *α*-methylacyl-CoA racemase, thyroid transcription factor-1, Epstein-Barr virus latent membrane protein-1, and CD30; in situ hybridization for human papillomavirus; and UroVysion fluorescence in situ hybridization. Williamson et al. [5] reported that the tumours they had identified from 34 patients were the latest series up to the time of publication of their paper and that the male to female ratio of the patients was 2.8 : 1 and the mean age of the patients was 70 years with the ages ranging from 54 years to 84 years. They had identified urothelial carcinoma in situ in 50% of the patients. On immunohistochemical staining 34*β*E12 was frequently positive in 75% of tumour cells of the patients, CK7 was frequently positive in 57% of tumour cells of the patients, and p63 was frequently positive in tumour cells of 53% of the patients; on the other hand thyroid transcription factor-1 and CD30 were consistently negative in the tumours. They had observed expression of p53 in 61% of the tumours, whereas CK20 staining was negative with the identification of weak positivity in one case only. UroVysion FISH had shown frequent chromosomal abnormalities similar to urothelial carcinoma. Williamson et al. [5] also found that, with regard to tumours that had concurrent urothelial, squamous, sarcomatoid, and glandular components, identical FISH abnormalities were observed. In situ hybridization for human papillomavirus and immunohistochemical staining for Epstein-Barr virus were negative in all the lesions they had studied. With regard to the outcome of the patients, Williamson et al. [5] reported that five of the patients who had pure or predominant lymphoepithelioma-like carcinoma tumours who had undergone transurethral resection of their tumours which was followed by chemotherapy were alive without any evidence of disease at 2 years to 5 years. On the contrary, 2 of the patients who had undergone the same type of treatment (transurethral resection of tumour followed by chemotherapy) with less than 50% lymphoepithelioma-like carcinoma morphology had died from the disease of distant metastasis. Williamson et al. [5] made the following conclusions:

- Urinary tract lymphoepithelioma-like carcinoma is a rare histological variant of urothelial carcinoma.
- The frequent presence of UroVysion FISH abnormalities, urothelial carcinoma in situ, and p53 positivity on immunohistochemical studies in cases of urinary tract lymphoepithelioma-like carcinoma would suggest a similar pathogenesis to high-grade invasive urothelial carcinoma.
- In contrast to the typical conventional urothelial carcinoma, CK20 is frequently negative with lymphoepithelioma-like carcinoma.
- Their findings would support the postulate that pure or predominant lymphoepithelioma-like carcinoma may be treated with transurethral resection and chemotherapy. Nevertheless, a large-scale study with long-term follow-up would be required to better understand the biological behaviour of lymphoepithelioma-like carcinoma of the urinary bladder.

It could be argued that perhaps the biological behaviour of lymphoepithelioma-like carcinoma of the prostate may be similar to lymphoepithelioma-like carcinoma variant of urothelial carcinoma but one cannot be certain whether this would be the case or not taking into consideration that very few cases of lymphoepithelioma-like carcinoma of the prostate gland had been reported and that its biological behaviour may not be known for certain. It therefore appear that a large-scale multicenter study with long-term follow-up would also be required in order to better understand the biological behaviour of lymphoepithelioma-like carcinoma of the prostate. It may also be argued that in view of the paucity of reported cases of lymphoepithelioma-like carcinoma of the prostate gland its true biological behaviour may not be known. Considering the fact that cases of primary lymphoepithelioma-like carcinomas of the prostate gland are rare there may be difficulties in establishing a multicenter trial; in that case hopefully if new cases of the disease encountered in the future are reported including pictures of the histological features of the disease and outcome following treatment a review of the literature in the future may enlighten clinicians regarding the biological behaviour of the disease.

## 5. Conclusions

Very few cases of primary lymphoepithelioma-like carcinoma of the prostate have been reported. Information gathered so far would indicate that primary lymphoepithelioma-like carcinomas of the prostate arise in carcinomas of the prostate gland that are in an advanced stage of the disease. In view of the scarcity of primary lymphoepithelioma-like carcinomas of the prostate there is no consensus regarding treatment options that would ensure good prognosis. Ideally cases of primary lymphoepithelioma-like carcinomas encountered by clinicians should be reported and they should be entered into a multicenter trial in order to ascertain the biological behaviour of the disease and to arrive at a consensus on the best treatment options that would improve the prognosis.

## Figures and Tables

**Figure 1:**
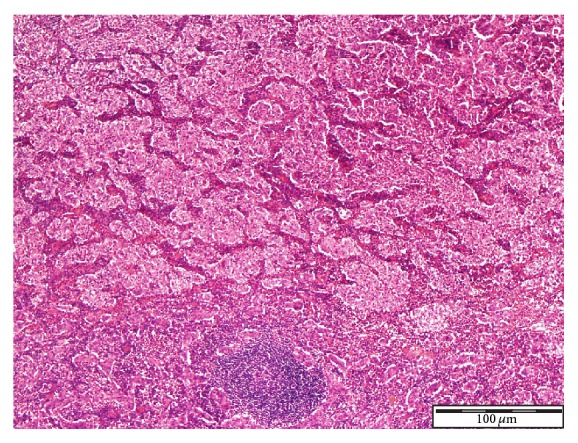
Lymphoepithelioma-like carcinoma of urinary bladder showing tumour cells admixed with dense inflammatory component Haematoxylin and Eosin staining ×4 magnification reproduced from [3] (http://dx.doi.org/10.7707/hmj.v6i3.245) with permission from Hamdan Medical Journal which states that it is in the interest of both author and HMJ that any future uses of the article include its HMJ publication details to ensure that other people cite it correctly. Copy Right © 2013 The Authors: The Journal Compilation © 2013 Sheikh Hamdan Bin Rashid Al Maktoum Award for Medical Sciences Copy Right is still retained by the original source and future use and reproduction of the figures would require copy right permission from the original source via Hamdan Medical Journal [because of the scarcity of primary lymphoepithelioma of the prostate there is no figure on the disease available to the author; hence the use of the figure is related to the urinary bladder].

**Figure 2:**
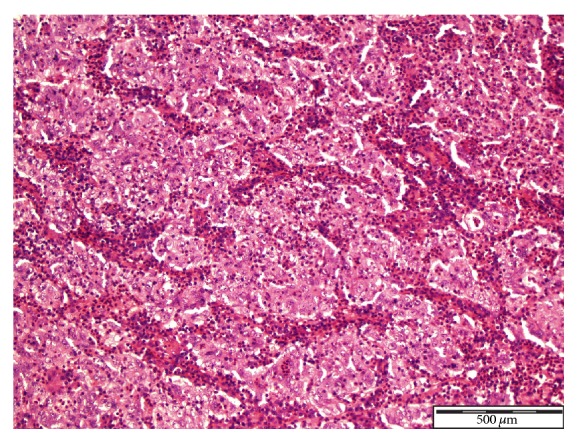
Lymphoepithelioma-like carcinoma of the urinary bladder showing neoplastic cells with syncytial growth pattern, surrounded by inflammatory cells Haematoxylin and Eosin staining ×10 magnification reproduced from [3] (http://dx.doi.org/10.7707/hmj.v6i3.245) with permission from Hamdan Medical Journal which states that it is in the interest of both author and HMJ that any future uses of the article include its HMJ publication details to ensure that other people cite it correctly. Copy Right © 2013 The Authors: The Journal Compilation © 2013 Sheikh Hamdan Bin Rashid Al Maktoum Award for Medical Sciences Copy Right is still retained by the original source and future use and reproduction of the figures would require copy right permission from the original source via Hamdan Medical Journal [because of the scarcity of primary lymphoepithelioma of the prostate there is no figure on the disease available to the author; hence the use of the figure is related to the urinary bladder].

**Figure 3:**
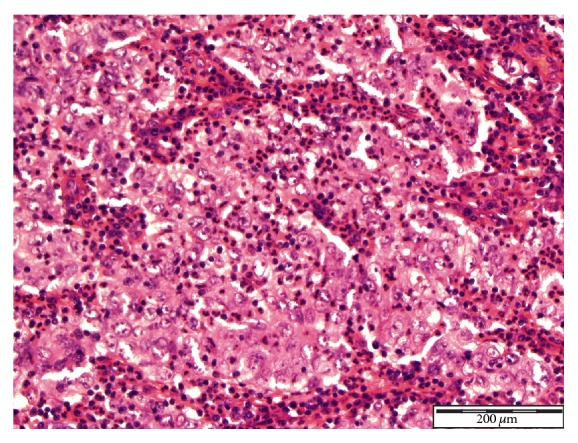
Lymphoepithelioma-like carcinoma of the urinary bladder showing neoplastic cells with indistinct cell borders. The tumour cells are showing eosinophilic cytoplasm, large vesicular nuclei, coarse chromatin, and prominent nucleoli Haematoxylin and Eosin staining ×20 magnification reproduced from [3] (http://dx.doi.org/10.7707/hmj.v6i3.245) with permission from Hamdan Medical Journal which states that it is in the interest of both author and HMJ that any future uses of the article include its HMJ publication details to ensure that other people cite it correctly. Copy Right © 2013 The Authors: The Journal Compilation © 2013 Sheikh Hamdan Bin Rashid Al Maktoum Award for Medical Sciences Copy Right is still retained by the original source and future use and reproduction of the figures would require copy right permission from the original source via Hamdan Medical Journal [because of the scarcity of primary lymphoepithelioma of the prostate there is no figure on the disease available to the author hence the use of the figure is related to the urinary bladder].
